# Persistence of the protective immunity and kinetics of the isotype specific antibody response against the viral nucleocapsid protein after experimental Schmallenberg virus infection of sheep

**DOI:** 10.1186/s13567-015-0260-6

**Published:** 2015-10-15

**Authors:** Antoine Poskin, Stephanie Verite, Loic Comtet, Yves Van der Stede, Brigitte Cay, Nick De Regge

**Affiliations:** CODA-CERVA, Operational Directorate Viral Diseases, Groeselenberg 99, 1180 Brussels, Belgium; CODA-CERVA, Coordination of Veterinary Diagnostics Epidemiology and Risk Analysis, Groeselenberg 99, 1180 Brussels, Belgium; ID Vet, Service développement, 310 Rue Louis Pasteur, 34790 Grabels, France; Department of Veterinary Virology, Parasitology and Immunology, Ghent University, Salisburylaan 133, 9820 Merelbeke, Belgium

## Abstract

Schmallenberg virus (SBV) is an *Orthobunyavirus* that induces abortion, stillbirths and congenital malformations in ruminants. SBV infection induces a long lasting seroconversion under natural conditions. The persistence of the protective immunity and the isotype specific antibody response upon SBV infection of sheep has however not been studied in detail. Five sheep were kept in BSL3 facilities for more than 16 months and subjected to repeated SBV infections. Blood was regularly sampled and organs were collected at euthanasia. The presence of SBV RNA in serum and organs was measured with quantitative real-time PCR. The appearance and persistence of neutralizing and SBV nucleoprotein (N) isotype specific antibodies was determined with virus neutralization tests (VNT) and ELISAs. The primo SBV infection protected ewes against clinical signs, viraemia and virus replication in organs upon challenge infections more than 15 months later. Production of neutralizing SBV specific antibodies was first detected around 6 days post primo-inoculation with VNT and correlated with the appearance of SBV-N specific IgM antibodies. These IgM antibodies remained present for 2 weeks. SBV-N specific IgG antibodies were first detected between 10 and 21 dpi and reached a plateau at 28 dpi. This plateau remained consistently high and no significant decrease in titre was found over a period of more than 1 year. Similar results were found for the neutralising antibody response. In conclusion, the SBV specific IgM response probably eliminates SBV from the blood and the protective immunity induced by SBV infection protects sheep against reinfection for at least 16 months.

## Introduction

Schmallenberg virus (SBV) is an *Orthobunyavirus* belonging to the family *Bunyaviridae* that emerged in continental Europe in 2011 [1]. It is a vector borne disease of ruminants and transmitted by small hematophagous insects called *Culicoides* [2]. Shortly upon infection, a viraemia develops that lasts four to 5 days and can coincide with a drop of milk production, diarrhoea and hyperthermia in adult cattle [3]. In sheep, clinical symptoms were never reported in adult animals under natural conditions and only few symptoms were described after experimental infection [1, 4]. Abortion, stillbirths and malformations can be observed in offspring upon SBV infection of pregnant cattle, sheep and goat [5].

*Orthobunyaviruses* have an RNA genome consisting of three segments named according to their size small (S), medium (M) and large (L). The S-segment encodes a non-structural protein (NSs) and a nucleoprotein (N), which is later associated with the genome in a ribonucleoprotein complex. The M-segment encodes two glycoproteins that are present in the viral envelope (Gn and Gc) and a non–structural protein (NSm). The L-segment encodes the RNA-dependent RNA polymerase (L) [6].

Commercial ELISA’s have been used to measure SBV-specific antibody production and they allowed detecting seroconversion in sheep 10 to 14 days post-infection (dpi) under experimental conditions [4, 7]. Despite a good concordance between ELISA and virus neutralisation tests (VNT), VNT has been shown to be more sensitive than the commercial ELISA [8–10]. Virus neutralisation test reported in literature were conducted with heat-inactivated serum (30 min at 56 °C) [8, 9, 11–16]. Heat-treatment of serum before VNT is a routine practice aiming to inactivate the complement system and is recommended by the OIE for SBV VNT [17].

Schmallenberg virus specific antibodies are known to persist at least 12–24 months in cattle after natural infection [13, 18]. Also in sentinel sheep herds it was observed that SBV-specific antibodies could last for at least 12 months [19]. Although these studies show that SBV-specific antibodies can last for a long time under natural conditions, one cannot exclude that multiple infections occurred, potentially at distinct moments over time. Another study suggested that naturally infected sheep were protected against clinical symptoms and induction of congenital malformations upon experimental reinfection [15].

Seen the fact that SBV was still circulating in Germany and the Netherlands in 2014 [20, 21] and the strong epidemiological similarity with Akabane virus, it is to be expected that SBV will persist in Europe [3,]. It is therefore important to obtain knowledge about the duration of the protective immunity and the development and persistence of the antibody response against this virus.

In this study, five ewes were maintained under experimental conditions during more than 1 year and subjected to SBV infection. The persistence of the protective immunity, the neutralizing antibody response and the kinetics of the isotype-specific antibody response against the SBV N protein were studied and quantified.

## Materials and methods

### Ethical statements

The experiments described hereafter were approved by the Ethical Committee of the IPH-VAR (Scientific Institute of Public Health-Veterinary and Agrochemical Research Centre, number of project: 121017-01).

### Animals, housing, inoculum and samples

This study was carried out with five “Mourerous” breed ewes between 14 and 17 months old at the moment of inoculation. All ewes were virologically and serologically negative for SBV at the start, as confirmed by quantitative real time polymerase chain reaction (qRT-PCR), VNT and ELISA. Ewes were housed together in a stable inside biosafety level 3 (BSL-3) facilities in which insect traps were used to monitor insects and confirm the absence of *Culicoides* midges throughout the experiment.

The inoculum used in this experience was an infectious bovine serum containing 2 × 10^3^ 50% tissue culture infectious doses/mL (TCID50/mL), as determined by end-point titration on baby hamster kidney cells that was provided by the Friedrich Loeffler Institute (Riems, Greifswald, Germany) [22]. The inoculum is known to successfully induce a viraemia followed by a seroconversion in sheep upon subcutaneous inoculation with one ml of the described inoculum [10]. Each animal was inoculated following the described route and dose at different time points. Four ewes (ewe 1 to 4) were inoculated at the beginning of the experiment (day 0). Ewe 3 and 4 received a booster inoculation 28 days post primo inoculation. The fifth ewe was kept as a control animal and was not primo inoculated nor received a booster. Thereafter all 5 ewes received a challenge inoculation more than 1 year after the primo inoculation (Table 1).

**Table 1:** **Experimental design**

Ewe	Inoculation	Booster	Challenge	Euthanasia
1	0	–	490	504
2	0	–	392	406
3	0	28	392	406
4	0	28	392	406
5	–	–	392	406

The table indicates the day of inoculation, booster and challenge infection, and the day of euthanasia for the five ewes used in this study. Ewe 1 and 2 did not receive a booster inoculation. Ewe 5 was not primo-inoculated nor boosted and kept as control until the challenge infection 14 days before euthanasia.

**Figure 1:**
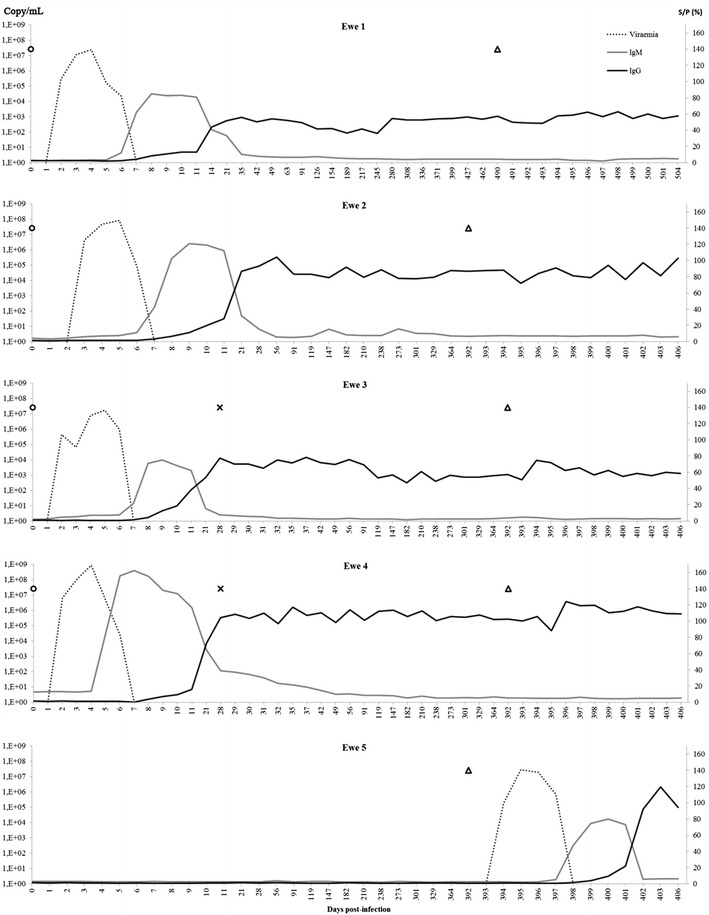
**RNAemia and SBV-N-specific antibody response induced by SBV inoculation in sheep**. RNAemia (copy/mL of serum) and isotype specific antibody response (IgM and IgG) against the SBV-N-protein in five ewes upon inoculation with SBV infectious serum under experimental conditions as measured with qRT-PCR and IgM ELISA and IgG ELISA respectively. The cut-off S/P value for the IgM ELISA was 17%, while for the IgG ELISA the cut-off value was 27%. Empty circles indicate the time points of SBV primo inoculations, crosses indicate the booster inoculations and empty triangles the challenge inoculations.

The five ewes were clinically examined, the rectal temperature was taken and the blood was collected after each inoculation daily during 1 week, then weekly for 1 month, and monthly thereafter (Figure 1). Serum was prepared from the blood by 15 min centrifugation at 3000 rpm and kept at −80 °C until further analysis. All ewes were euthanized 14 days after the challenge and the prescapularis lymph nodes, mesenteric lymph nodes, ovaries and spleen were collected during autopsy and stored at −80 °C until further analysis.

### SBV RNA detection in serum and organs with qRT-PCR

RNA extraction and qRT-PCR on blood and organs were conducted as previously described [11]. Briefly, total RNA was extracted from 140 µL of serum and eluted in 60 µL of elution buffer with the QIAamp Viral RNA Mini Kit following manufacturer’s instructions (Qiagen, Hilden, Germany). Before extraction, 100 µg of organ was homogenized in 1 mL of PBS with Stainless Steel Beads (Qiagen, Hilden, Germany) and shaken two times 2 min at 25 Hz with the TissueLyser (Qiagen, Hilden, Germany). The homogenate was subsequently centrifuged 5 min at 10 000 rpm. The total RNA was finally extracted from 100 µL of supernatant and eluted in 50 µL RNase free water with the RNeasy Mini Kit, following manufacturer’s instructions (Qiagen, Hilden, Germany). One negative control of extraction was added to each run of extraction.

The presence of SBV S-segment (forward primer: 5′-tcagattgtcatgccccttgc-3′; reverse primer: 5′-ttcggccccaggtgcaaatc-3′; probe: FAM-5′-ttaagggatgcacctgggccgatg GT-3′-BHQ1 [23]) and the presence of B-actin as internal control of extraction (forward primer: 5′-cagcacaatgaagatcaagatcatc-3′; reverse primer: 5′-cggactcatcgtactcctgctt-3′; probe: HEX-5′-tcgctgtccaccttccagcagcagatgt-3′-BHQ1 [24]) were detected in a duplex qRT-PCR carried out with the AgPath-ID one-step qRT-PCR kit (Life Technologies, California, USA) and measured with the LightCycler 480 Real-Time PCR system (Roche Applied Science, Indianapolis, USA). One positive control and one negative control of PCR amplification were added to each run of qRT-PCR. Cycle threshold values were converted into copy number/mL of serum and copy number/gr of organ using a standard curve consisting of successive ten-fold dilutions of an SBV S-segment RNA transcript that was added to each run of the qRT-PCR [10].

### Serology

Virus neutralization test was carried out as previously described [11] with the exception that all sera were analysed both without (VNTw/d) and with (VNTd) heat-inactivation of the complement system (30 min at 56 °C) before testing. Briefly, successive two fold dilutions of 50 µL of sera, from 1/2 to 1/256 onwards, were made in 50 µL of Dulbecco’s Modified Eagles Medium (Gibco, Life Technologies, Ghent, Belgium) supplemented by 1000 IU penicillin/mL, 50 µg/mL gentamicin (Gibco, Life Technologies, Ghent, Belgium) and 250 µg/mL amphotericin B (Gibco, Life Technologies, Ghent, Belgium) (DMEM) in Nunc Edge 96-well plates (Thermo scientific, Waltham, MA, USA). Fifty microliters of virus solution was added to each well and the plate let to incubate one hour at 37 °C. Thereafter 100 µL of DMEM with 10% foetal calf serum (Gibco, Life Technologies, Ghent, Belgium) and 4 × 10^6^ vero cells/mL were added to each well and the plate let to incubate at 37 °C and 5% CO_2_. After four days of incubation, the titre was determined as the reciprocal of the highest serum dilution in which no lysis plaques could be identified in the cell monolayer under the light microscope. Two positive controls and one negative control were added to each run of VNT. Sera were considered to be seronegative if the titre was lower than 4 (specificity 100%) [11].

The presence of SBV-N specific IgM antibodies was assayed with a capture IgM ELISA. ELISA plates coated with monoclonal anti-ruminant IgM antibodies, recombinant SBV-N-proteins and monoclonal horseradish peroxidase (HRP) labeled SBV-N specific antibodies were purchased from IdVet (Montpellier, France). Briefly, 10 µL of serum and 90 µL of dilution buffer were deposed in duplicate on the IgM ELISA plate and let to incubate 45 min at 37 °C. The wells were washed and 100 µL of the recombinant SBV N-protein solution was added to one of both replicates. One hundred microliter of the dilution buffer was added to the other replicate. After an incubation of 90 min at 37 °C, the wells were washed a second time and 100 µL of the ready-to-use conjugate was added into the wells and the plate was incubated 30 min at room temperature. After washing, 100 µL of the tetramethylbenzidine (TMB) substrate solution was added to the wells. After 15 min incubation in the dark at room temperature, the reaction was stopped by adding 100 µL of the stop solution. One positive and one negative control were added to each IgM ELISA plate. The control samples were defined and tested in preliminary experiments. The positive control sample consisted of pooled sera collected at 10, 11 and 14 dpi from one ewe inoculated with the same inoculum in a different experiment [25]. The negative control sample consisted of pooled sera collected from ewes in 2005. The optical density (OD) was red at 450 nm with Multiskan Ascent (Thermo Scientific, Waltham, MA, USA). The net OD value was calculated for each sample as (OD value in the replicate with the recombinant SBV N-protein solution—OD value in the replicate without the recombinant). The S/P percentage was calculated as (net OD value − OD negative control sample)/(OD positive control sample − OD negative control sample) × 100. The test was validated only if the ODpos >0.35 and the ODpos/ODneg >3. The cut-off value for SBV IgM was calculated based on the obtained values of 25 negative samples from the control ewe (ewe 5) collected before the first inoculation and eight sheep sera collected in 2005. The cut-off value was determined as the mean S/P value of the 33 negative samples +3 standard deviations. This interval represents 99.6% of the negative samples [26]. To evaluate the impact of heat-inactivation of serum (30 min at 56 °C) on SBV-N specific IgM detectability by ELISA, a selection of samples was tested in the IgM ELISA described above without (IgM ELISAw/d) and with (IgM ELISAd) heat-inactivation of the complement system.

The presence of SBV-N-specific IgG antibodies was measured with the ID Screen SBV Indirect test (IdVet, Montpellier, France) using an anti-multi-species IgG-horse radish peroxidase conjugate following manufacturer’s instructions [7]. The OD was measured as described above. The S/P percentage was calculated as (OD sample − OD negative control)/(OD positive control − OD negative control) × 100. The interpretation prescribed by the kit is as follows: negative if S/P ≤ 50%, doubtful if 50% < S/P ≤ 60% and positive if S/P > 60%. We also determined a cut-off value our self by testing, 50 field collected sera sampled before 2010. The cut-off value was determined as the mean S/P value of the 50 samples ± 3 × standard deviation. This interval represents 99.6% of the negative samples [26].

The concordance between the VNTd and the IgG ELISA was determined by dividing the number of samples for which the same qualitative result was obtained in VNTd and the IgG ELISA by the total number of samples analysed in both tests.

### Statistical analyses

The differences between the mean time needed to detect seroconversion with VNTw/d and VNTd, and the mean S/P value between IgM ELISAw/d and IgM ELISAd were statistically analysed with two samples paired *t* tests.

A linear mixed model was used (SAS Institute Inc., Cary, NC, USA) to estimate the mean effect of time with relation to the antibody titres and taking into account the correlation of the measurements within each sheep over time. The time effect was put as a fixed effect in the model and a random intercept was allowed meaning that there is an average ELISA titre in the sheep population after infection but that there is variability between the sheep. An autoregressive working correlation matrix (decreasing correlation for further time points) was selected based on the AIC (Aikake Information Criterion) value.

For all statistical tests, *P* values lower than 0.05 were considered to be statistically significant.

## Results

### Clinical impact

No significant clinical impact was noticed and the rectal temperature stayed within the normal range (38.3 °C ‒ 39.9 °C) throughout the experiment (data not shown) [27]. Ewes behaved normally, conserved a good appetite and no sign of diarrhoea was noticed until the end of the experiment.

### RNAemia

All ewes showed a comparable RNAemia that started on average 2.2 days post primo inoculation (95% CI 1.6–2.8) (Table 2). The RNAemia could be detected with qRT-PCR during a mean period of 4.6 days (95% CI 3.9–5.3). At the peak of RNAemia, the mean number of SBV RNA copies per ml of serum was 2 × 10^8^ (95% CI 0–6.5 × 10^8^). No viral RNA could be detected in the serum after the booster and the challenge inoculations. No RNAemia was found in the control ewe before its inoculation at the end of the experiment (Figure 1).

**Table 2:** **Period of RNAemia and first day of seroconversion upon SBV inoculation**

Ewe	RNAemia (first day)	RNAemia (last day)	VNTw/d	VNTd	ELISA IgM	ELISA IgG
1	2	7	8	9	7	14
2	2	7	6	9	7	21
3	2	7	6	6	6	11
4	3	7	6	9	7	21
5	2	6	5*	6	6	10

* 3 low positive results before first inoculation were not taken into account

Five ewes (ewe 1 to 5) were primo inoculated with SBV infectious serum at day 0 of the experiment. The numbers indicate the first and last day of RNAemia and the day of first seropositive result measured with virus neutralisation test without (VNTw/d) and with (VNTd) heat-inactivation of the serum. The first seropositive result measured with ELISA directed against the SBV N-protein for IgM (IgM ELISA, cut-off 17%) and IgG (IgG ELISA, cut-off 27%) are also given.

### Serology

**Figure 2:**
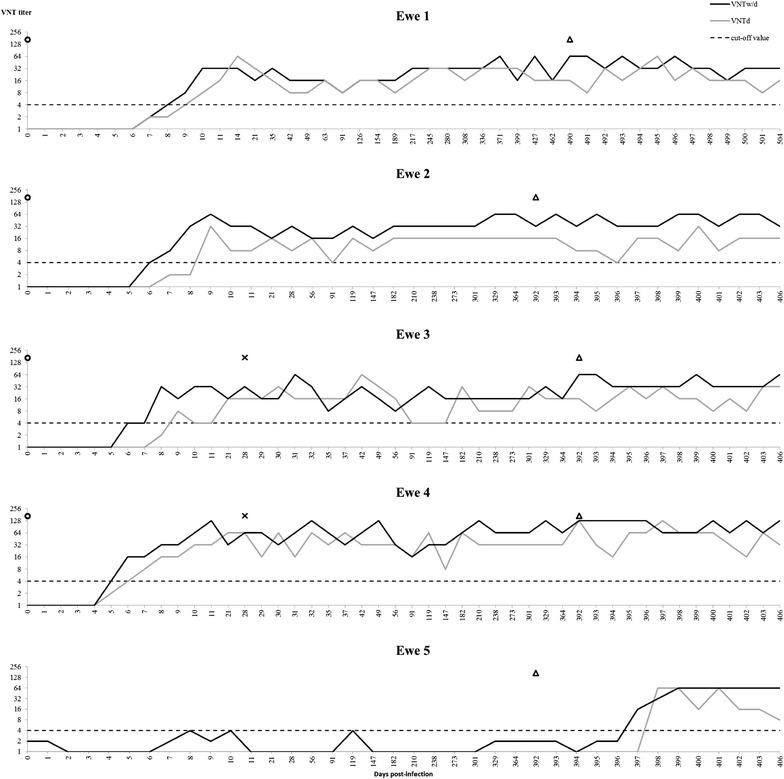
**Neutralizing antibody response induced by SBV inoculation in sheep.** SBV-specific neutralizing antibody production in five ewes inoculated with SBV infectious serum as measured with virus neutralization tests carried out without (VNTw/d) and with (VNTd) heat-inactivation of the complement system. Open circles indicate the time points of SBV inoculations, crosses indicate the time points of booster inoculations and open triangles indicate the time points of challenge inoculations.

Schmallenberg virus-specific neutralizing antibodies were first detected at 6.2 (95% CI 5.2–7.2) and 7.8 (95% CI 6.4–9.2) days post primo inoculation when determined by VNTw/d and VNTd, respectively. This difference was not significant, although the *P* value of 0.056 suggests that seroconversion is detected earlier when no heat-inactivation of the serum is performed. After seroconversion the titre fluctuated between 4 and 128 and stayed positive until the end of the experiment. No increase in antibody titre was observed after booster or challenge infections. When the serum was analysed without decomplementation, three false positive results were observed for the control ewe before its first inoculation at day 392 (days 8, 10 and 119) (Figure 2).

The cut-off value for the SBV-N-specific IgM ELISA was calculated to be 17%. The mean period for the first detection of SBV-N-specific IgM was 6.6 days (95% CI 6.1–7.1) post primo inoculation (Table 2) and SBV-N-specific IgM remained detectable for about two weeks. No production of SBV-N-specific IgM was detected after booster and challenge infections in ewes 1–4 (Figure 1). No significant differences were found between S/P values obtained with SBV-N-specific IgM ELISA in sera that were tested without and with heat-inactivation of the complement system.

The cut-off value of the SBV-N-specific IgG ELISA was determined to be at 27%. The first positive SBV-N-specific IgG antibody titre was measured between 10 and 21 days post primo inoculation in all ewes (Table 2). Since blood was only collected weekly after 11 days post primo inoculation, it is not possible to pinpoint the seroconversion date more precisely. The S/P values reached a plateau at 28 dpi. Applying a mixed linear model to the data obtained for ewe 1–4 between 28 and 392 dpi predicted an average decrease of 0.0204% in ELISA titre per day. This estimate was non-significantly different from 0 (*P* = 0.1734; F value = 3.16), indicating that no significant decrease over time in S/P values was found. Furthermore, no effect was observed after the booster or challenge inoculations with the exception of a small and temporal (1–4 days) decrease in the SBV-N specific IgG antibodies (Figure 1).

An overall concordance of 92% was found between the VNTd and the IgG ELISA. When the cut-off value prescribed by the manufacturer for the IgG ELISA is used, ewe 1 only became seropositive after the challenge inoculation, making that the concordance is reduced to 69% in that case).

### Necropsy

No lesions were noticed at necropsy in any of the ewes. Schmallenberg virus RNA was only detected in the four organs collected from the control ewe (ewe 5), which was inoculated only once at 14 days before euthanasia: mesenteric lymph nodes (8.7 × 10^8^ copies/gr of organ), spleen (2 × 10^8^ copies/gr), prescapularis lymph node (1.6 × 10^7^ copies/gr) and ovary (3.7 × 10^6^ copies/gr).

## Discussion

Several studies have shown that natural SBV infection and vaccination induces a protective immunity in cattle and sheep [13, 15, 28–30] but the duration of this induced immunity was not studied in detail. This work evaluated the persistence of the protective immunity induced by SBV infection of SBV naïve ewes over a period of more than 1 year under experimental conditions. This experiment furthermore analysed the kinetics of the isotype specific antibody response against the viral N-protein.

It was demonstrated that the immunity that develops after a single SBV infection of sheep protects the animal against clinical signs, RNAemia and virus replication in target organs upon reinfection for at least 15 months. This period probably lasts even longer since the infection was associated with a persistent neutralizing antibody response that did not significantly decrease during that time period. Although not specifically addressed in this study, it seems reasonable to hypothesize that this induced immunity capable of preventing RNAemia upon reinfection will also protect against transplacental virus transmission. Epidemiological data have shown that the massive spread of SBV among ruminants during the first outbreak season, as evidenced by a very high seroprevalence of SBV-specific antibodies, correlated with a strongly decreased incidence of congenital malformations during the next season despite a renewed virus circulation [18]. Another interesting consequence of the observation that no renewed RNAemia could be detected after the booster or challenge inoculations is that the short lasting RNAemia of 4 to 5 days after primo infection is the only time frame in which *Culicoides* midges can take an infectious blood meal and transmit the virus towards new hosts.

The long term protection and the fast and persistent SBV-specific antibody response after a primo infection of sheep indicate further that naïve new-born lambs represent on the one hand an appropriate target for a sentinel monitoring system to detect renewed circulation of the virus, but are on the other hand also the most important target population for vaccination. Although the long term protective effect of vaccination still has to be confirmed, it seems the best way to protect livestock against SBV related disease for a long period of time [28, 29].

Our results show that the decrease of SBV RNA in serum coincides with the appearance of SBV-specific neutralizing antibodies as detected by VNT and SBV-N-specific IgM antibodies as found in ELISA, suggesting that SBV-specific IgM plays an important role in the clearance of the virus from the circulation. The observation that the VNT detects seroconversion around the same time as the SBV-N-specific IgM ELISA furthermore strongly suggests that the detection of SBV-specific IgM by VNT explains the higher sensitivity of VNT to detect seroconversion than the commercially available ELISA detecting SBV-N IgG as was suggested before [10]. Since it has been described for other *Orthobunyaviruses* that also glycoproteins Gn and Gc can be the target of a neutralizing antibody response besides the N-protein [31], it will be interesting to study the antibody response against both other proteins in the future.

It was observed that seroconversion could be detected earlier by VNT in sera that had not been heat-inactivated. This might be explained by the fact that heat treatment of sera could result in a (partial) destruction of IgM [32, 33]. The heat-inactivation of the sera had however no effect on the SBV-N-specific IgM detection in ELISA. Another possible explanation is that a part of the (early) SBV-specific antibodies require complement factors to fully resort their neutralizing effect as has been shown before for the antibody response against several other viruses [34–37]. This seems to be supported by the fact that VNT titres obtained for untreated sera were often higher than those of heat-inactivated sera, also on time points when SBV-specific IgM was no longer present. Although omitting heat treatment seems to allow detection of seroconversion earlier by VNT, the occurrence of false positive results in the control ewe in our experiment suggests keeping the heat-inactivation of sera for VNT in place.

The decrease of SBV-N specific IgM at about 2 weeks post infection is associated with an increase in SBV-N specific IgG. The stable and long lasting (neutralizing) IgG response is in line with VNT results obtained after infection of sheep with the related AKAV [38] and suggests a role of these antibodies in the long term protection against SBV reinfection. Also the cell-mediated immunity might play a role in the long term protection, but was not addressed in this study. Its importance has already been suggested based on the lymphohystiocytic infiltration of the white and grey matters in SBV affected foetuses [39–41] and it was suggested to be part of the protective immunity induced by SBV vaccination [28], but its contribution remains to be elucidated.

Our results with the SBV-N-specific indirect IgG ELISA highlight that one should be critical when defining the infection status of an animal which has an S/P value close to the cut-off described by the manufacturer. Although the cut-off of this commercial IgG ELISA was chosen based on results of many serum samples and a ROC analysis, ewe 1 from this experiment would have been considered as negative or doubtful till the moment of the challenge infection. It seems therefore advisable that doubtful samples are confirmed in the VNTd. ELISA test are however less time consuming and more suitable for testing of a large amount of samples and it would also be interesting to evaluate the sensitivity of competition ELISAs that are currently available since these tend to be more sensitive.

Since IgA is known to be mostly present in mucous secretions and only at low levels in sheep blood [42], the kinetic of this particular antibody isotype was not investigated. It could be more interesting to test the presence of SBV-specific IgA in nasal swabs or milk in the future.

In conclusion, it was shown that a single SBV infection of sheep induces a protective immunity that prevents against clinical signs, RNAemia and virus transmission to organs upon SBV reinfection more than 15 months later. The initial clearance of the virus from the blood coincided with the appearance of SBV-specific neutralizing antibodies and SBV-N-specific IgM around one week post-infection. Between 2 and 3 weeks post-infection, an SBV-N-specific IgG response appeared that reached a plateau at 4 weeks post-infection and remained stable for more than 1 year. It furthermore showed that despite the fact that omitting heat-inactivation of sera allows to detect seroconversion about 1 day earlier, the risk for false positive results suggests to keep decomplementation in place when performing VNT.
